# Influence of different noninvasive oxygenation support devices on tidal volume

**DOI:** 10.1186/s13613-023-01200-2

**Published:** 2023-11-25

**Authors:** Anne-Fleur Haudebourg, Tommaso Maraffi, Samuel Tuffet, Philippe Le Corvoisier, Armand Mekontso Dessap, Guillaume Carteaux

**Affiliations:** 1 Service de Médecine Intensive Réanimation, Assistance Publique-Hôpitaux de Paris, CHU Henri Mondor - Albert Chenevier, 51, Avenue du Maréchal de Lattre de Tassigny, 94010 Créteil Cedex, France; 2 Faculté de Santé, Groupe de Recherche Clinique CARMAS, Université Paris Est-Créteil, 94010 Créteil, France; 3 INSERM U955, Institut Mondor de Recherche Biomédicale, 94010 Créteil, France; 4 Service de Réanimation Adultes Et Surveillance Continue, Centre Hospitalier Intercommunal Créteil, 40, Avenue de Verdun, 94000 Créteil, France; 5 Centre d’Investigation Clinique 1430, Assistance Publique-Hôpitaux de Paris, CHU Henri Mondor - Albert Chenevier, 51, Avenue du Maréchal de Lattre de Tassigny, 94010 Créteil Cedex, France

**Keywords:** Respiratory failure, Noninvasive ventilation, Continuous positive airway pressure, High-flow oxygen through nasal cannula, Oxygen therapy, Tidal volume, Patient self-inflicted lung injury

## Abstract

**Background:**

Multiple devices are available for noninvasive oxygenation support, including non-rebreather oxygen mask (O_2_-mask), high-flow oxygen through nasal cannula (HFNC), continuous positive airway pressure (CPAP), mask noninvasive ventilation (Mask-NIV) and helmet NIV (Helmet-NIV). As tidal volume is a key determinant of efficacy and safety during ventilatory support, we assessed whether it was influenced by the type of noninvasive oxygenation device.

**Methods:**

A bench study using a manikin with a realistic face connected to a lung simulator was performed. Six conditions were assessed: no device, O_2_-mask, HFNC, CPAP, Mask-NIV and Helmet-NIV. Three respiratory mechanics were simulated (normal, obstructive, restrictive), at three simulated efforts (low, moderate, respiratory distress). Flow was recorded at the lung simulator inlet and mouth pressure into the manikin mouth. The same devices were evaluated on healthy volunteers with tidal volume assessed by electrical impedance tomography (EIT).

**Results:**

Tidal volume was significantly influenced by oxygenation devices in bench model. As compared to O_2_-mask, HFNC and CPAP delivered significantly lower tidal volumes (440 ± 352 mL, 414 ± 333 mL and 377 ± 297 mL, respectively), while Mask-NIV or Helmet-NIV were associated with significantly higher tidal volumes (690 ± 321 mL and 652 ± 366 mL, respectively). Tidal volume was strongly correlated with the specific effect of each device on mouth pressure during inspiration: HFNC and CPAP were characterized by a negative PTPmouth (− 0.3 [− 0.8 to − 0.2] and − 0.7 [− 2.2 to − 0.5] cmH_2_O.sec/cycle, respectively), while Helmet-NIV and Mask-NIV were associated with a positive PTPmouth (4.5 [4.1–4.6] and 6.1 [5.9–7.1] cmH_2_O.sec/cycle, respectively). Tidal volume was also significantly influenced by oxygenation devices in healthy volunteers, with similar tidal volumes between O_2_-mask and CPAP (644 [571–764] and 648 [586–770] mL) but higher with HFNC, Mask-NIV and Helmet-NIV (819 [609–918], 1110 [661–1305] and 1086 [833–1243] mL).

**Conclusions:**

Tidal volume is significantly influenced by noninvasive oxygenation support devices, with a strong correlation with the pressure variation generated into the mouth during inspiration. NIV was associated with the highest tidal volumes and CPAP with the lowest ones. Clinical studies are needed to clarify the clinical implications of these effects.

**Supplementary Information:**

The online version contains supplementary material available at 10.1186/s13613-023-01200-2.

## Introduction

Multiple devices for noninvasive oxygen delivery during acute hypoxemic respiratory failure (AHRF) are available: non-rebreather oxygen mask (O_2_-mask), high-flow oxygen through nasal cannula (HFNC) [1], continuous positive airway pressure (CPAP) [2], and noninvasive ventilation (NIV), which can be conducted using different interfaces [oro-nasal mask (Mask-NIV) [3–5] or helmet (Helmet-NIV)] [6]. There are conflicting data about their respective clinical effects and to date, no recommendation has been made concerning their choice [7]. Several factors may drive the clinical outcome when using a noninvasive technique to avoid intubation. In recent years, a growing body of evidence pointed out the potential deleterious effect of high tidal volumes related to high stress during NIV for de novo AHRF [8–11], raising concern about the risk of patient self-inflicted lung injury (P-SILI) [12] during noninvasive strategies. The tidal volume is not measurable at the bedside with most oxygenation support devices, impeding further exploration of the potential hazard of high tidal volume.

Tidal volume may depend on patient’s characteristics (i.e., respiratory drive and respiratory mechanics), the physiological response to the application of the noninvasive device, and some of its specific mechanical effects. The relationship between these different parameters at each moment “*t*” during inspiration is described by the equation of motion of the respiratory system:

1 $${\text{Ptot}}_{{({\text{t}})}} \, = \,{\text{Paw}}_{{({\text{t}})}} \, + \,{\text{Pmus}}_{{({\text{t}})}} \, = \,{\text{P}}0\, + \,{\text{Raw }} \times {\text{V}}_{{({\text{t}})}}{\prime} \, \, + \,{\text{VT}}_{{({\text{t}})}} /{\text{C}}_{{{\text{RS}}}} .$$

Ptot: total pressure in the respiratory system, Paw: airway pressure, Pmus: muscular pressure generated by inspiratory muscles contraction, P0: end-expiratory pressure, Raw: airways resistance, V’: inspiratory flow, VT: tidal volume, C_RS_: respiratory system compliance.

During AHRF, some physiological consequences of the application of O_2_-mask, HFNC, CPAP, Mask-NIV and Helmet-NIV have been reported [6, 8, 13–15], but their respective mechanical effects have not been compared so far.

The purpose of this study was therefore to compare mechanical effects (i.e., pressure, flow, and volume) generated by different noninvasive devices available to deliver oxygen in standardized conditions.

## Materials and methods

This study was designed as a bench study and then a healthy volunteer study.

### Bench study

#### Devices tested

Six devices were tested (Additional file 1: Fig. S1):

1. Non-rebreather oxygen mask with an oxygen flow at 15 L/min (O_2_-mask);
2. HFNC (Optiflow; Fisher & Paykel Healthcare, Auckland, New Zealand) using a flow of 60 L/min and a fraction of inspired O_2_ (FiO_2_) of 0.6. The manikin’s mouth was kept closed during HFNC;
3. Boussignac CPAP (Vygon, Ecouen, France) with an oxygen gas flow of 30 L/min, titrated to obtain a continuous pressure of 10 cmH_2_O. Boussignac CPAP was applied using an oronasal mask (FreeMotion, Fisher & Paykel Healthcare, Auckland, New Zealand);
4. Helmet CPAP using a StarMed CA-Star helmet (Intersurgical, Berkshire, UK) and a mechanical PEEP valve. We used a gas flow of 80 L/min in order to obtain a CPAP of 10 cm H_2_O, and a FiO_2_ of 0.6;

For the sake of clarity, data from the Boussignac and Helmet CPAP were pooled and analyzed as a whole (labeled as CPAP). A comparison of Boussignac and Helmet CPAP showing no significant difference is available in the ESM (Additional file 4: Table S1).

5- Mask-NIV with an oronasal mask (FreeMotion, Fisher & Paykel Healthcare, Auckland, New Zealand); and
6- Helmet-NIV delivered with a StarMed NIV Helmet (Intersurgical, Berkshire, UK).

Both NIV interfaces were applied using a R860 Carescape ventilator (GE Healthcare, Fairfield, CT, USA) with the NIV mode engaged. The PEEP level was set at 5 cm H_2_O, the pressure support level at 10 cm H_2_O, the inspiratory trigger at 1 L/min, the pressurization slope at 50 ms and the FiO_2_ at 0.6.

With each interface, particular care was taken to avoid leaks.

#### Bench model

Each device was tested using a manikin head with realistic upper airways (RespiSim^®^ Manikin, IngMar Medical, Pittsburg, PA, USA) whose trachea was connected to an Active Servo Lung 5000 test lung (ASL5000®; IngMar Medical, Pittsburg, PA, USA) (Additional file 2: Fig. S2). Three different respiratory mechanics were simulated: normal (compliance: 60 mL/cm H_2_O, resistance: 5 cm H_2_O/L/s), obstructive (compliance: 60 mL/cm H_2_O, resistance: 20 cm H_2_O/L/s) and restrictive (compliance: 30 mL/cm H_2_O and resistance: 5 cm H_2_O/L/s). Each respiratory mechanics was tested at three simulated respiratory efforts: low (inspiratory muscle pressure [Pmus] of 5 cm H_2_O), moderate (inspiratory Pmus of 10 cm H_2_O), and respiratory distress (inspiratory Pmus of 20 cm H_2_O, and expiratory Pmus of 10 cm H_2_O), using a simulated respiratory rate of 25 breaths per minute. Details about ASL 5000 parameters during the inspiratory and expiratory phases are presented in the ESM (Additional file 4: Table S2). Each combination of respiratory mechanics and effort was also tested without any device (NO DEVICE). Tidal volumes obtained in each experimental condition were also referred to an “average male patient” of 175 cm of height (the average height in France according to the national institute of statistics, INSEE [16]), and a predicted body weight (PBW) of 70 kg. In addition to comparisons between all the devices, the tidal volume in HFNC and CPAP obtained with a simulated inspiratory effort of 10 cm H_2_O was compared to that obtained in NIV with a simulated respiratory effort reduced by half, in order to replicate the reported physiological effects of pressure support [10, 13, 17].

#### Recordings

Flow was recorded using a pneumotachograph AC137A-1 (Biopac Systems, Goleta, CA, USA) inserted between the manikin trachea and the test lung at the ASL inlet. Airway pressure was recorded in the manikin mouth using a differential pressure transducer TSD160D (Biopac Systems, Goleta, CA, USA). All signals were recorded at 2000 Hz using an analog/numeric data-acquisition system (MP150, Biopac systems, Goleta, CA, USA) and stored in a computer for subsequent analysis with AcqKnowledge software version 5.0 (Biopac systems, Goleta, CA, USA).

Definitions of signals assessed are described in Additional file 3: Fig. S3. Tidal volume (Vt) was defined as the area under the positive flow curve. Peak inspiratory flow was the maximum flow achieved during the inspiratory phase. Peak mouth pressure was the extreme value (positive or negative) of mouth pressure during inspiratory time. PEEP was measured as the mean pressure recorded during the last 200 ms of the expiration in the manikin mouth. The inspiratory mouth pressure–time product (PTPmouth) was defined as the area under the mouth pressure curve from the onset of the simulated inspiratory effort to the end of the inspiratory time.

### Healthy volunteer study

The study was designed and conducted at Henri Mondor University Hospital and approved by the French Ethics Committee CPP (comité de protection des personnes) Sud-Ouest et Outre Mer III (2020-A03088-31).

#### Subjects’ recruitment

Subjects were included in the study if they were healthy, with no underlying respiratory disease, and aged more than 18 years. Exclusion criteria included pregnancy, lack of social support, legal protective measures, and the presence of cardiac implantable devices like pacemakers or implantable cardioverter defibrillators. Every participant in the study provided written informed consent before being recruited.

#### Experimental design and sequence of interventions

Healthy volunteers were consecutively subjected to 10-min ventilation sessions with each of the devices previously described in the bench part of the study except Helmet CPAP, in half sitting position, with a FiO_2_ of 0.21 for HFNC, Boussignac CPAP and NIV. Both NIV interfaces were applied using a Monnal T60 ventilator (Air Liquide Medical Systems, Fairfield, CT, USA). The PEEP level was set at 5 cm H_2_O with Mask-NIV and 10 cm H_2_O with Helmet-NIV [6, 18, 19]. The pressure support level was adjusted at 10 cm H_2_O, the inspiratory trigger at 1 L/min and the pressurization slope at 50 ms.

#### Recordings

Subjects were investigated by electrical impedance tomography (EIT, Enlight 1800, Timpel, Sao Paulo, Brazil) using a belt containing 32 electrodes placed around the patient’s chest at the fifth or sixth intercostal space. In order to obtain tidal volume with all tested devices, we calculated for each subject a conversion coefficient *k* between chest impedance tidal variations and tidal volume measured by integration of the flow signal during Mask-NIV (with a pressure support level of 10 cm H_2_O and a PEEP of 5 cm H_2_O), using a pneumotachograph (AC137A-1, Biopac Systems, Goleta, CA, USA) inserted on the ventilator circuit between the mask and the Y-piece. The conversion coefficient *k* was then applied to EIT data in order to calculate tidal volume under each experimental condition [14]. The impedance variations were averaged over 20 cycles during a stable period at the end of the 10-min recording for each condition.

We also evaluated the tolerance of each device with a numerical discomfort scale, scored from 0 to 10, where 0 represented a very comfortable device and 10 an extremely uncomfortable device.

### Statistical analysis

Statistical analysis was performed with SigmaPlot 12.0 and Statistical Package for the Social Sciences (version 16.0, SPSS, Chicago, IL, USA). Normality of the distribution was evaluated with the Shapiro–Wilk test. Data are reported as means and standard deviation for continuous normally distributed variables and median and 25–75th interquartile range for continuous non-normally distributed variables. Continuous variables were compared with Student’s t or Mann–Whitney rank sum tests as appropriate. Categorical variables were compared with the Chi (Χ^2^) test or Fisher’s exact test as appropriate. Different devices were compared using an ANOVA for repeated measures or the Friedman test; correction for multiple testing was performed using the Benjamini–Hochberg method. Pearson correlation analysis was used to assess correlation between tidal volumes and mouth pressure–time products generated by oxygenation devices in various respiratory mechanics and simulated respiratory effort conditions. Statistical significance was reached when two-tailed p value was less than 0.05.

## Results

### Bench study

#### Mechanical effect of the oxygenation device on tidal volume

**Fig. 1:**
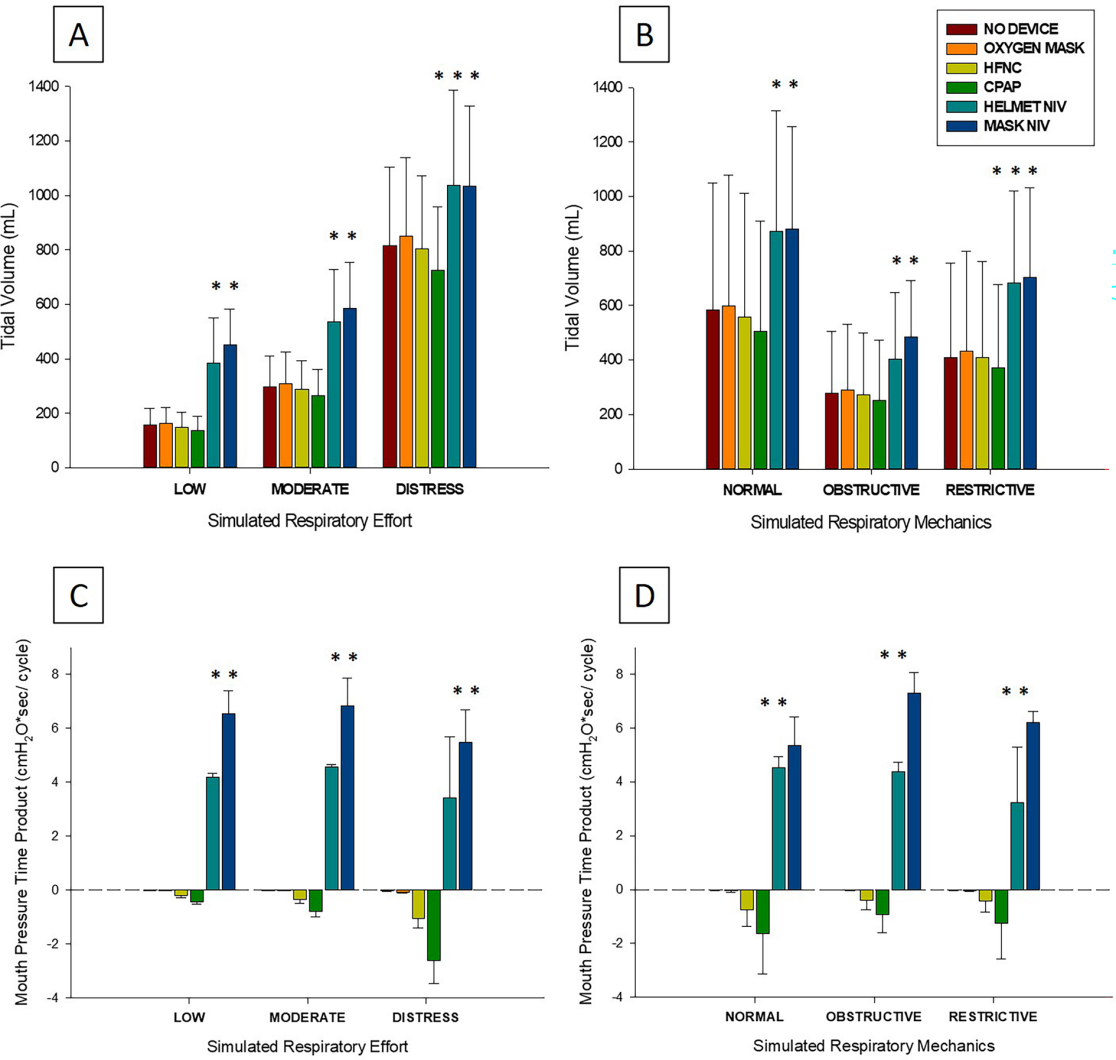
Effect of each device on tidal volume and mouth pressure–time product in bench model. **A** and **C** Represent the effect of each device on tidal volume (**A**) and mouth pressure–time product (**C**) according to the simulated respiratory effort. A low respiratory effort denotes an inspiratory muscle pressure of 5 cmH_2_O; a moderate respiratory effort denotes an inspiratory muscle pressure of 10 cmH_2_O; a distress denotes an inspiratory muscle pressure of 20 cmH_2_O combined with an expiratory muscle pressure of 10 cmH_2_O. Panels B and D represent the effect of each device on tidal volume (**B**) and mouth pressure–time product (**D**) according to the respiratory mechanics. *p* < 0.001 for both overall comparisons on one-way ANOVA. * denotes *p* < 0.05 *vs.* O_2_-mask within each respiratory effort and respiratory mechanics. HFNC: high-flow oxygen through nasal cannula; CPAP: continuous positive airway pressure; NIV: noninvasive ventilation

In comparison to the O_2_-mask, both HFNC (*p =* 0.005) and CPAP (*p =* 0.005) were significantly associated with lower tidal volumes, whereas Mask-NIV (*p =* 0.002) and Helmet-NIV (*p =* 0.002) were significantly associated with higher tidal volumes (Fig. 1A, B and Table 1).

**Table 1:** Ventilatory variables across devices, all mechanics and efforts pooled, in bench model

	No device	O_2_-mask	HFNC	CPAP	Helmet-NIV	Mask-NIV	*p*
V_T_, mL	423* (± 340)	440 (± 352)	414* (± 333)	377* (± 297)	652* (± 366)	690* (± 321)	< 0.001
V_T_, mL/kg PBW	6.0 (± 4.9)	6.3 (± 5.0)	5.9* (± 4.8)	5.4* (± 4.2)	9.3* (± 5.2)	9.9* (± 4.6)	< 0.001
PTPmouth, cmH_2_O.s/cycle	− 0.0 [− 0.0 to − 0.0]	− 0.0 [− 0.0 to − 0.0]	− 0.3* [− 0.8 to − 0.2]	− 0.7* [− 2.2 to − 0.5]	4.5* [4.1–4.6]	6.1* [5.9–7.1]	< 0.001
Peak inspiratory pressure, cmH_2_O	− 1.1 [− 3.6 to − 0.5]	− 1.1 [− 3.8 to − 0.5]	− 0.1* [− 3.1 to 0.6]	8.1* [3.9–9]	15.5* [13.2–18]	15.4* [15.2–19.3]	< 0.001
PEEP, cmH_2_O	0.1 (± 0.2)	0.1 (± 0.2)	1.5* (± 0.4)	10.3* (± 0.5)	5.9* (± 0.7)	6.2* (± 0.9)	< 0.001
Peak inspiratory flow, ml/s	470 [284 – 985]	493 [298–1035]	466* [307–827]	437* [261–898]	1338* [1058–1709]	1018* [797–1380]	< 0.001

O_2_-mask: non-rebreather oxygen mask; HFNC: high-flow oxygen through nasal cannula; CPAP: continuous positive airway pressure; Helmet-NIV: noninvasive ventilation with a Helmet; Mask-NIV: noninvasive ventilation with an oronasal mask; V_T_: tidal volume; PBW: predicted body weight; PTP: pressure–time product; PEEP: positive end-expiratory pressure

Data are presented as mean (standard deviation) or as median [25th–75th interquartile range]. *P* value presented is overall comparison on one-way ANOVA

*p < 0.05 *vs.* O_2_-mask

**Fig. 2:**
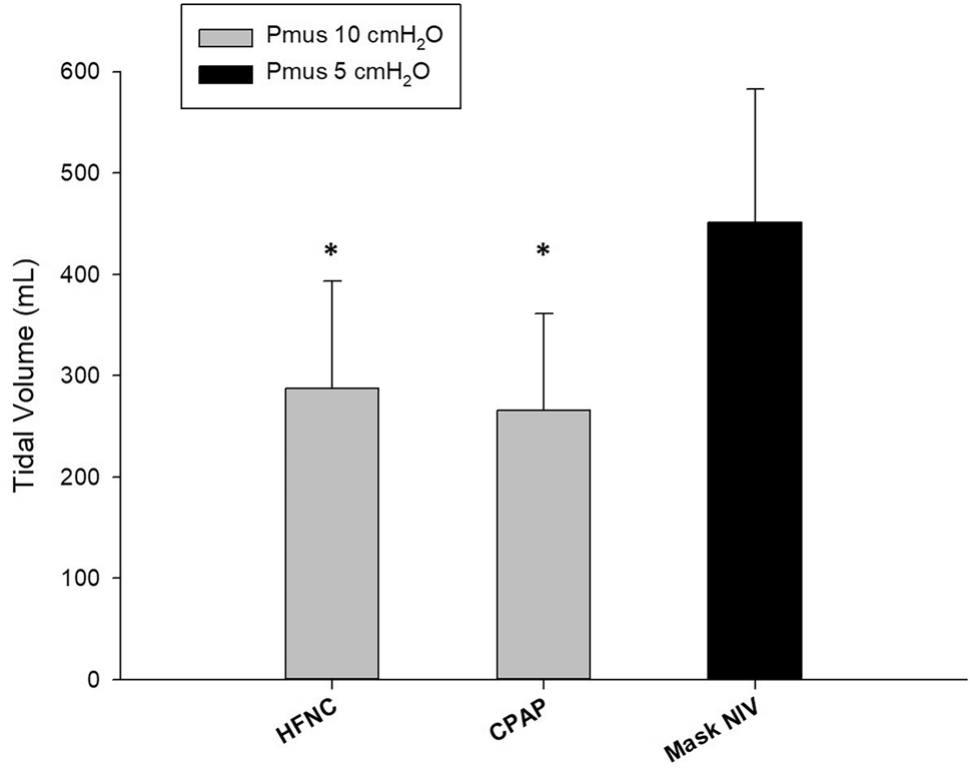
Comparison of Mask-NIV with low inspiratory effort and HNFC or CPAP with moderate inspiratory effort. Even when dividing by two the inspiratory effort, the tidal volume generated with the use of NIV conducted with an oro-nasal mask (Mask-NIV) remained largely higher than the tidal volumes observed with the use of high-flow oxygen through nasal cannula (HFNC) or continuous positive airway pressure (CPAP). * denotes p < 0.05 *vs.* mask NIV. Pmus: inspiratory muscle pressure

When referred to an “average” male patient of 70 kg of predicted body weight, tidal volumes ranged from 6 ± 4.9 mL/kg for no device to 9.9 ± 4.6 mL/kg for Mask-NIV. Mask-NIV delivered significantly higher tidal volumes than HFNC and CPAP even when the simulated respiratory effort was twice lower with the use of Mask-NIV as compared to HFNC and CPAP (451 ± 132 mL *vs.* 288 ± 106 mL and 266 ± 95 mL, respectively, p < 0.001, Fig. 2).

#### Mechanical effect of the oxygenation device on mouth pressure

**Fig. 3:**
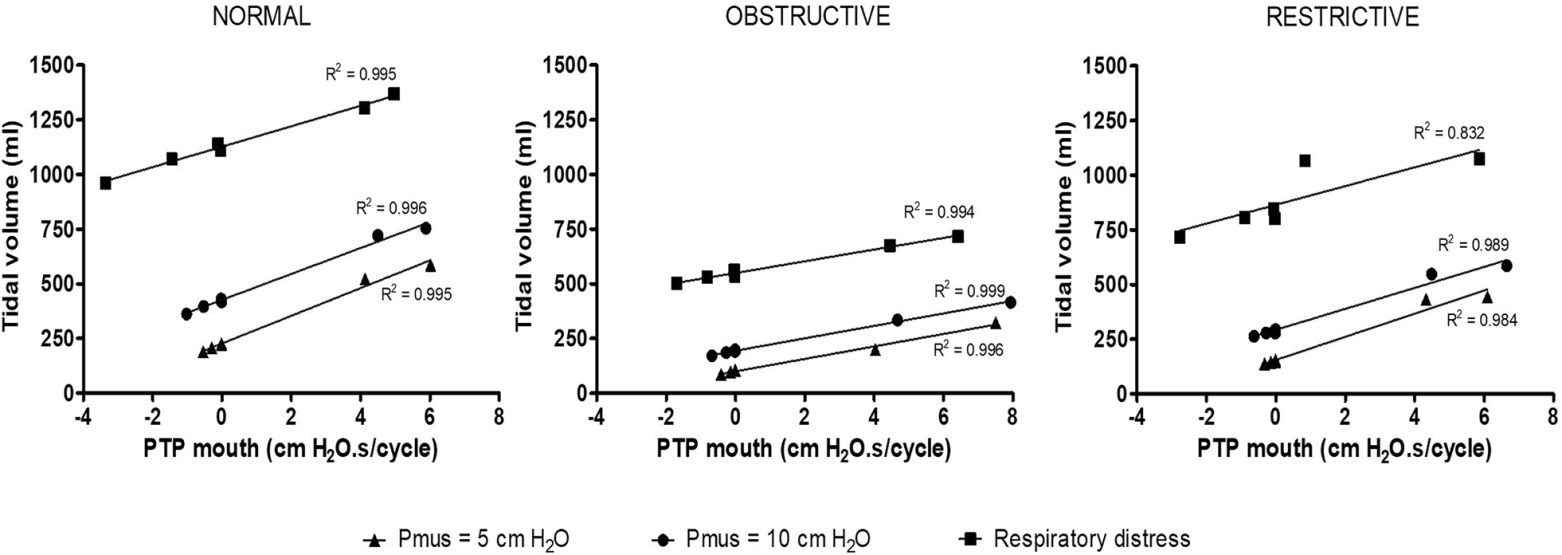
Correlation between mouth pressure–time product and tidal volume in bench model. Pearson correlation between mouth pressure–time product and tidal volume. Lines represent the linear regression slopes. Triangles, dots and squares represent individual data with low respiratory effort, moderate respiratory effort and respiratory distress, respectively. PTP: pressure–time product

The pressure generated into the mouth, assessed using its pressure–time product (PTPmouth), was significantly influenced by the devices tested (Fig. 1C, D and Table 1). HFNC and CPAP were characterized by negative PTPmouth values while Mask-NIV and Helmet-NIV were characterized by positive PTPmouth values. In each combination of simulated respiratory mechanics and effort, there was a strong correlation between the PTPmouth and the resulting tidal volume (Fig. 3). A positive end-expiratory pressure of 1.5 ± 0.4 cm H_2_O was observed during the application of HFNC when the manikin’s mouth was kept closed. This effect disappeared (PEEP = 0.3 ± 0.2 cm H_2_O) when the manikin’s mouth was left open.

### Healthy volunteer study

**Fig. 4:**
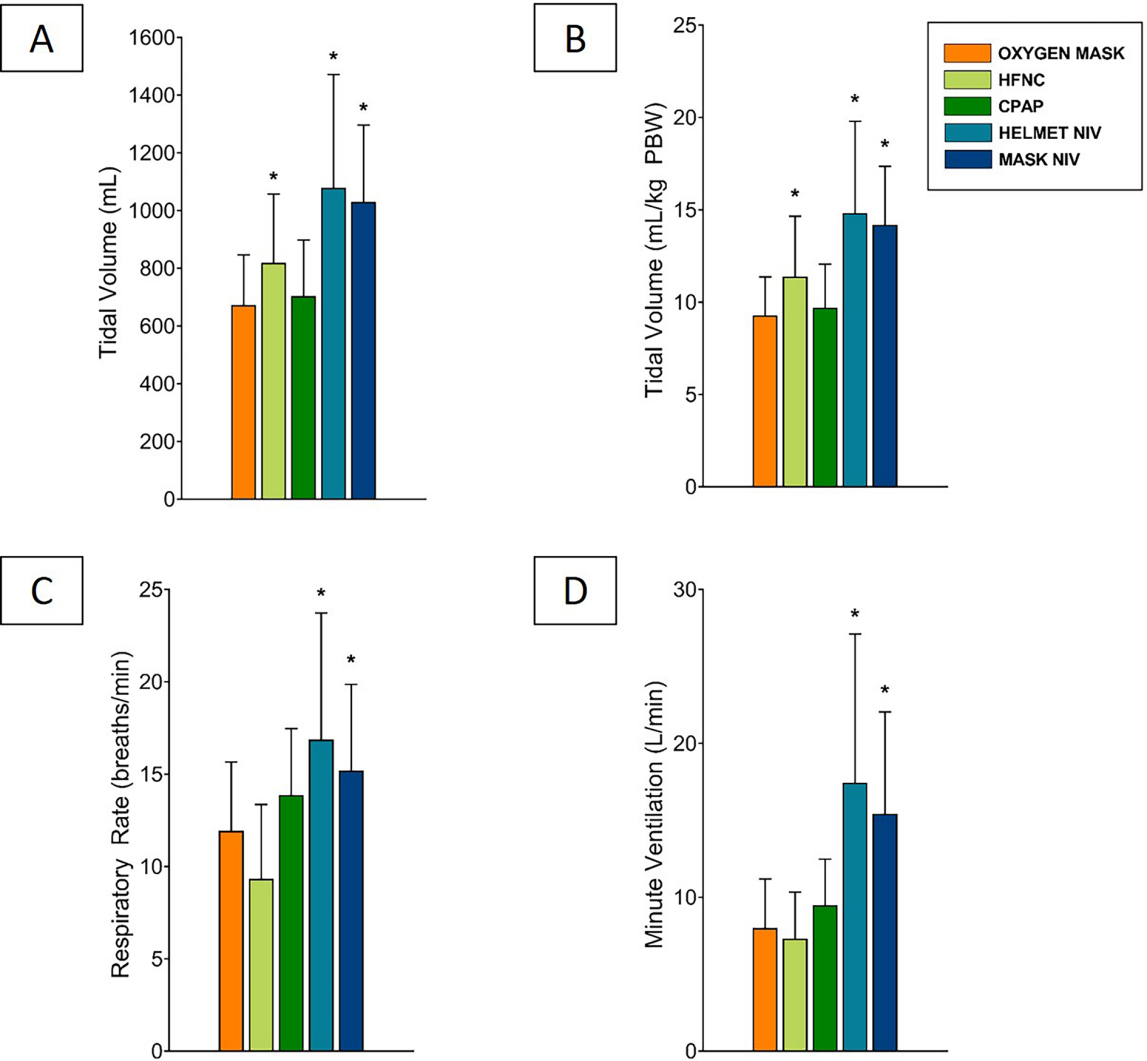
Physiological variables of healthy volunteers according to each device. Column bars represent the effect of each device on tidal volume (**A**, **B**), respiratory rate (**C**) and minute ventilation (**D**). p < 0.0001 for overall comparisons on one-way ANOVA. * denotes p < 0.05 *vs.* O_2_-mask. HFNC: high-flow oxygen through nasal cannula; CPAP: continuous positive airway pressure; NIV: noninvasive ventilation; PBW: predicted body weight

The 15 participants’ baseline characteristics are listed in Additional file 4: Table S3. Compared to O_2_-mask, the tidal volume was similar during CPAP, whereas it increased significantly with HFNC, Mask-NIV and Helmet-NIV (Fig. 4A and Table 2). Minute ventilation during HFNC and CPAP was similar than during O_2_-mask, whereas Mask-NIV and Helmet-NIV were associated with significantly higher minute ventilation (Fig. 4D and Table 2).

**Table 2:** Physiological variables of healthy volunteers according to each device

	O_2_-mask	HFNC	CPAP	Helmet-NIV	Mask-NIV	*p*
V_T_, mL	644 [571–764]	819* [609–918]	648 [586–770]	1110* [661–1305]	1086* [833–1243]	< 0.0001
V_T_, mL/kg PBW	8.8 [7.8–10.2]	10.7* [9.6–12.4]	9.0 [8.3–10.8]	14.3* [10.3–18.0]	14.0* [11.4–17.4]	< 0.0001
RR, breaths/min	11 [9–15]	8 [6–11]	14 [11–16]	18* [15–20]	14* [11–20]	< 0.0001
MV, L/min	6.6 [5.7–10.8]	5.9 [5.0–9.2]	9.8 [7.3–11.1]	14.3* [11.2–23.1]	13.0* [11.4–15.2]	< 0.0001
Delta EELV *vs* O_2_-mask, mL	–	+ 214 [374–711]	+ 705 [951–1286]	+ 939 [1115–1417]	+ 226 [364–635]	< 0.0001
Anterior ventilation, %	47 [43–51]	46 [44–52]	43 [40–53]	51* [45–55]	51* [47–58]	0.0001
Discomfort scale	2 [1–3]	4* [3–6]	3* [2–6]	7* [6–8]	4* [4, 5]	< 0.0001

O_2_-mask: non-rebreather oxygen mask; HFNC: high-flow oxygen through nasal cannula; CPAP: continuous positive airway pressure; Helmet-NIV: noninvasive ventilation with a Helmet; Mask-NIV: noninvasive ventilation with an oronasal mask; V_T_: tidal volume; PBW: predicted body weight; RR: respiratory rate; EELV; end-expiratory lung volume

Data are presented as median [25th–75th interquartile range]. P value presented is overall comparison on one-way ANOVA

*p < 0.05 *vs.* O_2_-mask

## Discussion

The main finding of our study was that different devices currently used to deliver noninvasive oxygenation support have a significant influence on tidal volume. The lowest tidal volumes were observed with the use of CPAP and HFNC in bench model of spontaneous breathing and with the use of CPAP and O_2_-mask in healthy volunteers. The highest tidal volumes were observed during NIV in a bench model and in healthy volunteers. We also observed, in the bench model, that different devices had radically different effects on the pressure generated into the mouth during inspiration and that there was a strong correlation between mouth pressure and tidal volume.

### Device, Pmouth and tidal volume

**Fig. 5:**
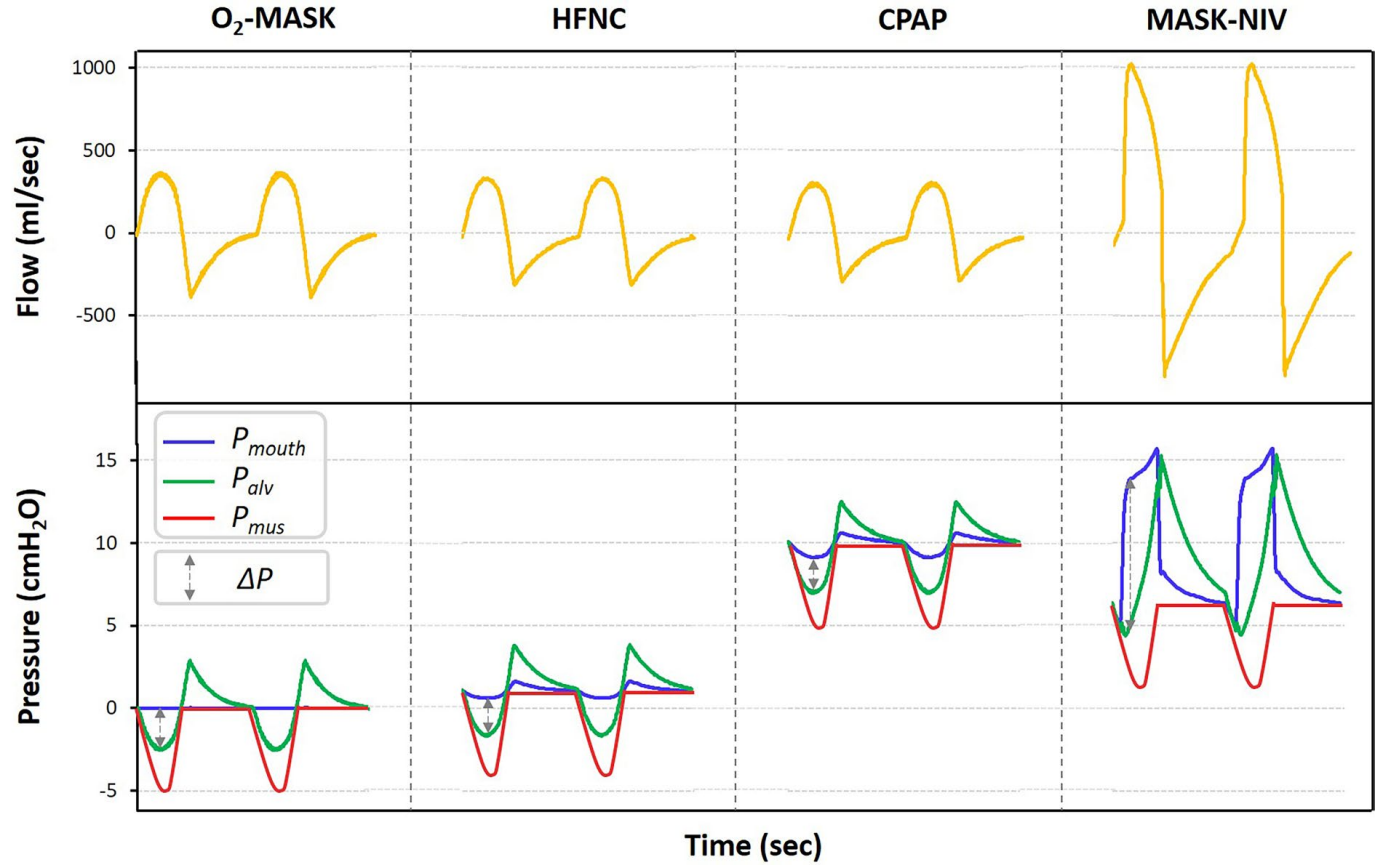
Effect of various oxygenation devices on inspiratory driving pressure in bench model. Inspiratory flow depends on the pressure difference between the mouth pressure and the alveolar pressure, which results from the negative pressure produced by respiratory muscles contraction during spontaneous breathing. As compared to the use of a non-rebreather oxygen mask (O_2_-MASK), during which the mouth pressure (Pmouth) remained constant, there was a drop in Pmouth with the use of high-flow oxygen through nasal cannula (HFNC) and continuous positive airway pressure (CPAP), decreasing the inspiratory driving pressure (ΔP), and rise in Pmouth with the use of NIV conducted with an oro-nasal mask (Mask-NIV), leading to an important increase in the inspiratory driving pressure. Alveolar pressure was computed using the following equation: Palv = Paw−(Raw x V′). O_2_-mask: non-rebreather oxygen mask; HFNC: high-flow oxygen through nasal cannula; CPAP: continuous positive airway pressure; Mask-NIV: noninvasive ventilation with oronasal mask; Palv: alveolar pressure; Pmouth: mouth pressure; Pmus: muscle pressure; ∆P: driving pressure

Interestingly, in the bench model, we found that devices that deliver a PEEP without additional inspiratory pressure support (HFNC and CPAP) were associated with negative inspiratory mouth pressure swings that were significantly more pronounced than with no device or O_2_-mask. Inspiratory flow depends on the pressure difference between the mouth pressure (entrance pressure) and the alveolar pressure, which results from the negative pressure produced by respiratory muscles contraction during spontaneous breathing. Without a device or with one that does not generate any positive pressure (like the O2-mask), (i) the inspiratory effort will reduce the alveolar pressure, as depicted by the solid green line in Fig. 5; however (ii) it will not affect the mouth pressure (constant solid blue line in Fig. 5). This is because the mouth pressure equates to the barometric pressure, which remains unaltered by the inspiratory effort. With devices producing continuous positive pressure (CPAP or HFNC), the starting mouth pressure during inspiration shifts from barometric to the PEEP value. The inspiratory effort will then: (i) still cause the reduction in alveolar pressure (under a constant effort model); and (ii) also lead to a decrease in mouth pressure, especially if the device does not compensate quickly or adequately to sustain a constant pressure [20]. This decrease will be even more pronounced with increased effort. In the example given in Fig. 5 (bench model with normal respiratory mechanics and low effort), the maximum pressure gradient during inspiration (represented by the dotted gray arrow) is lower with HFNC or with CPAP than with O2-mask or no device. As the pressure gradient driving inspiratory flow is smaller, peak inspiratory flow is lower and therefore tidal volume is lower. This effect has already been described during CPAP in bench studies [21–23], and depends on the CPAP device [23]. Furthermore, in a physiological study involving ten patients in acute hypoxemic respiratory failure, L’Her et al*.* reported a tidal volume reduction with the use of CPAP as compared to oxygen alone or NIV [13]. We noted a similar effect in the bench study during CPAP and HFNC (closed mouth condition) when a PEEP was present. In healthy volunteers, HFNC did not reduce tidal volume. However, minute ventilation was similar between HFNC and O2-mask (5.9 L/min [5.0–9.2] vs. 6.6 L/min [5.7–10.8], *p =* 0.91) because of low respiratory rate with HFNC (8 breaths/min [6–11]), with an expiration described as difficult by most participants (discomfort scale at 4 [3–6] in median with HFNC), which may be due to an increase in expiratory resistance [20].

### Clinical implications

To assess the clinical relevance of our bench model, we referred to measured tidal volumes of an average patient, with a predicted body weight (PBW) of 70 kg. The mean tidal volumes observed with the use of devices delivering no additional inspiratory pressure were around 5–6 ml/kg of PBW, while they increased above 9 ml/kg of PBW when NIV was applied, consistent with what has been reported so far in the clinical setting [8, 9]. These results are consistent with literature reporting that high tidal volumes during Mask-NIV in de novo hypoxemic acute respiratory failure are frequent despite careful titration of pressure support level [8, 9]. As there is increasing concern that high tidal volumes during spontaneous or assisted ventilation might worsen lung injury and promote patient self-inflicted lung injury (P-SILI) [12], the knowledge of the respective mechanical effect of each noninvasive oxygenation support device on tidal volume in standardized conditions may be of clinical interest.

### Strengths and limitations

Our bench study was conducted on a high-fidelity model, simulating different respiratory mechanics at different levels of respiratory effort. This allowed assessing the specific mechanical effects of each oxygenation support device using standardized characteristics. The results were then validated on healthy volunteers. Our study also has multiple limitations. First, our model was not able to automatically simulate the physiological response to an increase in inspiratory assistance in terms of muscle unloading, nor the response to PEEP. The rationale behind the use of NIV in acute respiratory failure lies in relieving patient distress and dyspnea by transferring part of the inspiratory work from the patient to the ventilator, thus reducing patients’ respiratory drive [24]. However, we explored on a bench model the effect on tidal volume while dividing by two the respiratory effort during NIV, in accordance to what has been described in patients experiencing acute hypoxemic respiratory failure [10, 13, 17]. Even after having divided by two the respiratory effort, tidal volumes generated with Mask-NIV were still almost twice higher than those recorded with HFNC or CPAP. Second, the compliance of the respiratory system did not change with changes in end-expiratory lung volume; therefore, any potential recruitment effect of PEEP on respiratory mechanics was not taken into account. However, NIV (with oro-nasal mask or helmet), which delivers an additional inspiratory pressure support, was associated with the highest PTPmouth and therefore the highest tidal volumes, whatever the respiratory effort or mechanics. Furthermore, the results were consistent with our findings in healthy volunteers, despite an increase in end-expiratory lung volume. Thirdly, the relatively short recording duration (10 min) in healthy volunteers may have been insufficient to allow a complete adaptation of ventilatory command. Nevertheless, studies in patients have demonstrated that the ventilatory responses to inspiratory unloading by pressure support ventilation occur rapidly, often within a range of seconds to minutes [25]. In our study, conducting the analyses at the end of the recording period likely promoted stabilization of respiratory control.

## Conclusions

In conclusion our data showed that tidal volume was significantly influenced by noninvasive oxygenation support devices. The magnitude and direction of tidal volume change observed with each device were strongly correlated with its effect on the pressure variation generated into the mouth during inspiration. NIV was thus associated with the highest tidal volumes and CPAP with the lowest ones in a bench model and in healthy volunteers. Clinical studies in patients with acute hypoxemic respiratory failure are needed to scrutinize the clinical implications of these effects.

### Supplementary Information

**Additional file 1: Figure S1.** Devices tested. All devices were tested on the RespiSim^®^ Manikin connected to an ASL5000 test lung (IngMar Medical, Pittsburg, PA, USA). Care was taken to avoid leaks. **A** Non-rebreather oxygen mask (O_2_-mask). **B** Boussignac CPAP; **C** Helmet CPAP; data from the Boussignac and Helmet CPAP were pooled and analyzed as a whole (CPAP). **D** High-flow oxygen through nasal cannula (HFNC). **E** NIV using an oro-nasal mask (Mask-NIV); F: NIV using a helmet (Helmet-NIV).

**Additional file 2: Figure S2.** Representation of the experimental setup. Each device was tested using the RespiSim^®^ Manikin connected to an ASL5000 test lung (IngMar Medical, Pittsburg, PA, USA). Flow was recorded using a pneumotachograph inserted between the manikin and the test lung. “Mouth pressure was recorded using a differential pressure transducer inserted into the manikin mouth. All signals were recorded using an analog/numeric data-acquisition system (MP150, Biopac systems, Goleta, CA, USA) and stored in a computer for subsequent analysis with AcqKnowledge software (Biopac systems, Goleta, CA, USA).

**Additional file 3: Figure S3.** Main signals analysis: representative tracings from respiratory cycles during CPAP and Mask-NIV. Flow was recorded using a pneumotachograph inserted between the manikin and the test lung. Mouth pressure was recorded using a differential pressure transducer inserted into the manikin mouth. Tidal volume was defined as the area under the positive flow curve (hatched area). The inspiratory time (**a**) was defined as the time during which the flow was positive. Peak inspiratory flow (**b**) was the maximum flow recorded during inspiration. Peak mouth pressure (**c**) was the extreme value (positive or negative) of mouth pressure during inspiratory time. PEEP (**d**) was measured as the mean pressure recorded during the last 200 ms of the expiration at the manikin mouth. The inspiratory mouth pressure–time product (PTPmouth) was the area under the mouth pressure curve from the onset of the simulated inspiratory effort to the end of the inspiratory time (dotted area).

**Additional file 4: Table S1.** Comparison between the BOUSSIGNAC CPAP and the HELMET CPAP, all mechanics and efforts pooled. **Table S2.** Active Servo Lung 5000 test lung settings during each experimental setup. **Table S3.** Characteristics of the Participants (*n* = 15).

## Data Availability

The datasets used and analyzed during the current study are available from the corresponding author on reasonable request.

## Abbreviations

AHRF: Acute hypoxemic respiratory failure
CPAP: Continuous positive airway pressure
EELV: End-expiratory lung volume
EIT: Electrical impedance tomography
HFNC: High-flow oxygen through nasal cannula
NIV: Non-invasive ventilation
Mask-NIV: Mask non-invasive ventilation
Helmet-NIV: Helmet non-invasive ventilation
P-SILI: Patient self-inflicted lung injury
PEEP: Positive end-expiratory pressure
PTP: Pressure–time product
Vt: Tidal volume
