# Supplementary figures and images for: Influence of different noninvasive oxygenation support devices on tidal volume
Source: Ann Intensive Care. 2023 Nov 25;13:116. doi: 10.1186/s13613-023-01200-2 (PMC10676331; doi:10.1186/s13613-023-01200-2)

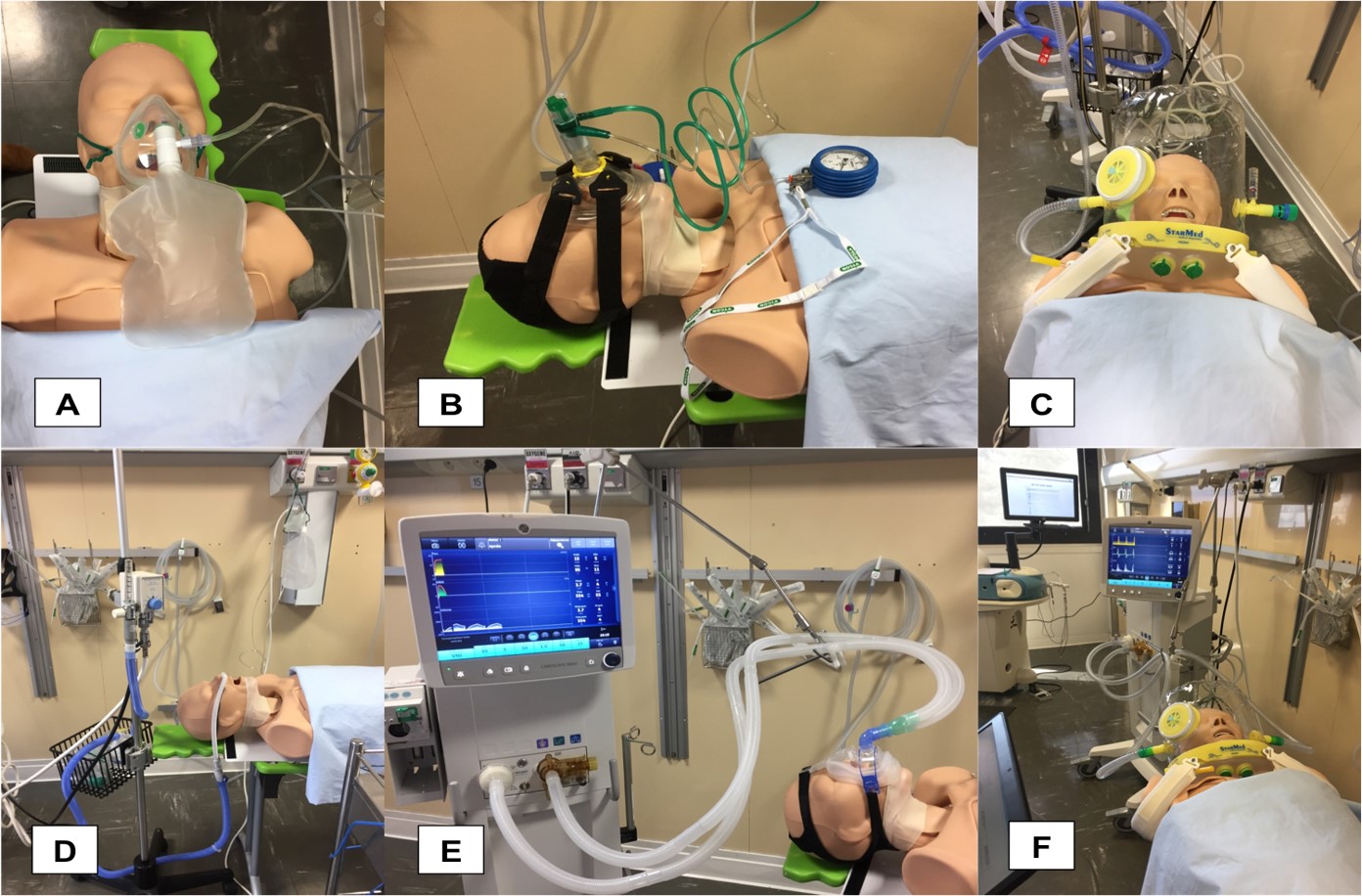

Supplement: Supplementary file 1 — Additional file 1: Figure S1. Devices tested. All devices were tested on the RespiSim® Manikin connected to an ASL5000 test lung (IngMar Medical, Pittsburg, PA, USA). Care was taken to avoid leaks. A Non-rebreather oxygen mask (O2-mask). B Boussignac CPAP; C Helmet CPAP; data from the Boussignac and Helmet CPAP were pooled and analyzed as a whole (CPAP). D High-flow oxygen through nasal cannula (HFNC). E NIV using an oro-nasal mask (Mask-NIV); F: NIV using a helmet (Helmet-NIV). [file 13613_2023_1200_MOESM1_ESM.jpg]

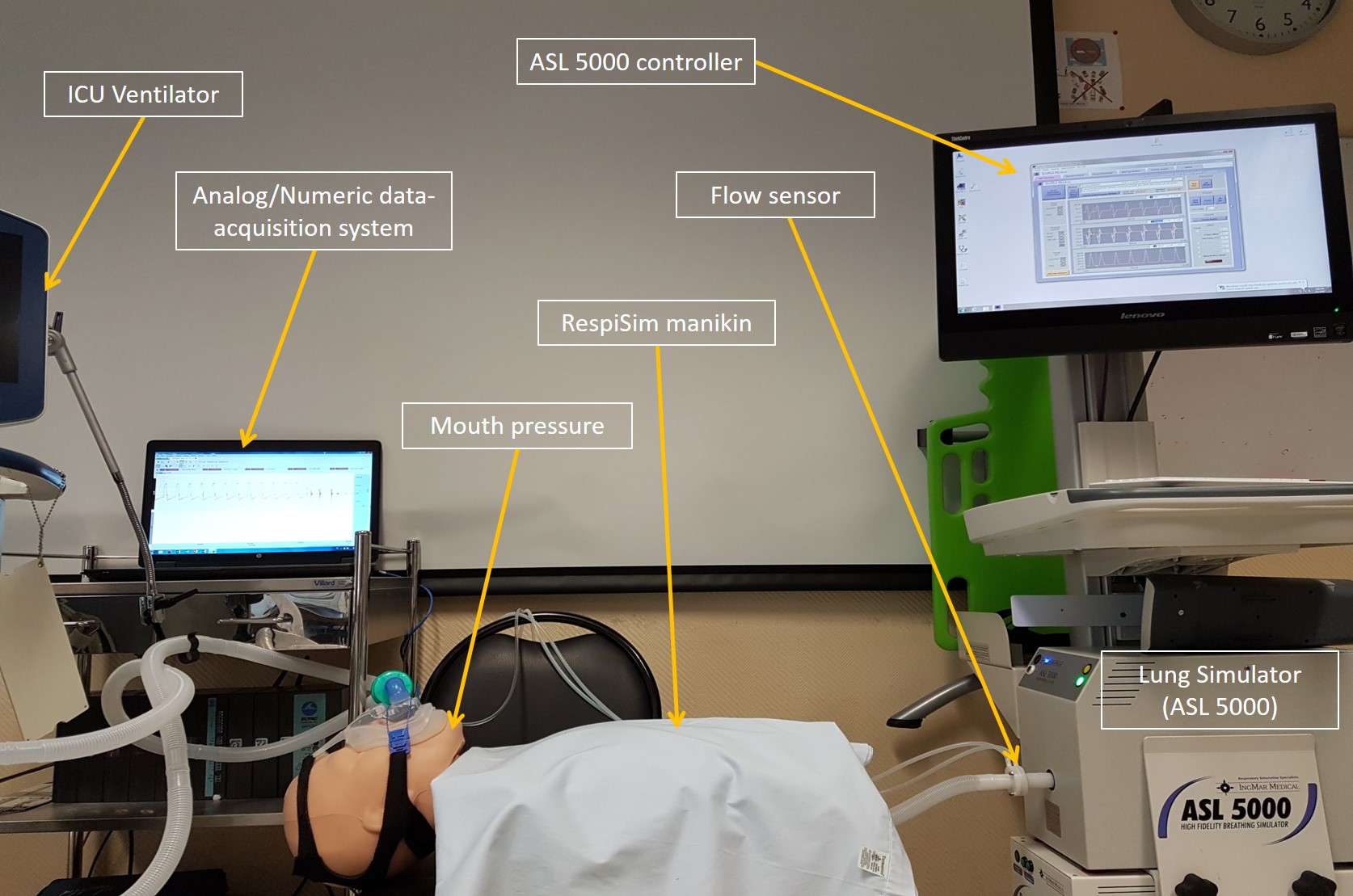

Supplement: Supplementary file 2 — Additional file 2: Figure S2. Representation of the experimental setup. Each device was tested using the RespiSim® Manikin connected to an ASL5000 test lung (IngMar Medical, Pittsburg, PA, USA). Flow was recorded using a pneumotachograph inserted between the manikin and the test lung. “Mouth pressure was recorded using a differential pressure transducer inserted into the manikin mouth. All signals were recorded using an analog/numeric data-acquisition system (MP150, Biopac systems, Goleta, CA, USA) and stored in a computer for subsequent analysis with AcqKnowledge software (Biopac systems, Goleta, CA, USA). [file 13613_2023_1200_MOESM2_ESM.jpg]

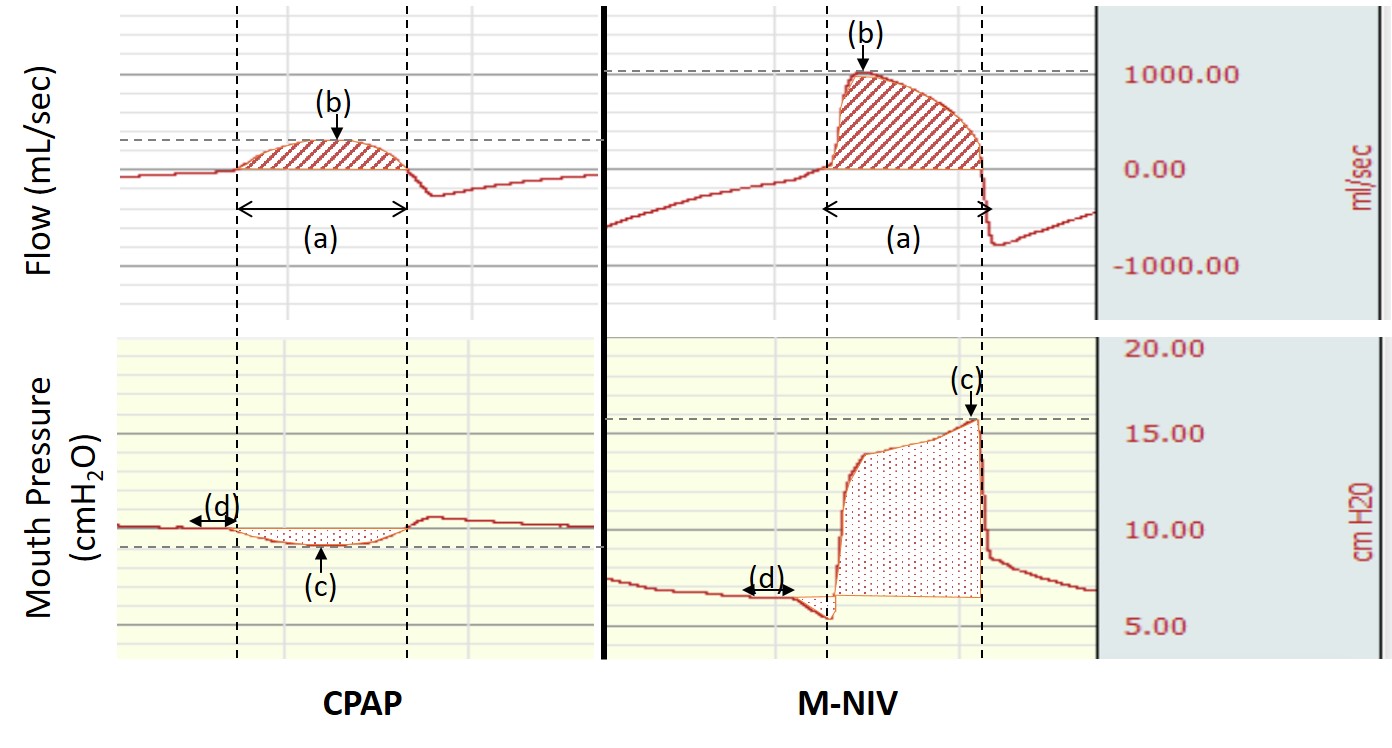

Supplement: Supplementary file 3 — Additional file 3: Figure S3. Main signals analysis: representative tracings from respiratory cycles during CPAP and Mask-NIV. Flow was recorded using a pneumotachograph inserted between the manikin and the test lung. Mouth pressure was recorded using a differential pressure transducer inserted into the manikin mouth. Tidal volume was defined as the area under the positive flow curve (hatched area). The inspiratory time (a) was defined as the time during which the flow was positive. Peak inspiratory flow (b) was the maximum flow recorded during inspiration. Peak mouth pressure (c) was the extreme value (positive or negative) of mouth pressure during inspiratory time. PEEP (d) was measured as the mean pressure recorded during the last 200 ms of the expiration at the manikin mouth. The inspiratory mouth pressure–time product (PTPmouth) was the area under the mouth pressure curve from the onset of the simulated inspiratory effort to the end of the inspiratory time (dotted area). [file 13613_2023_1200_MOESM3_ESM.jpg]
