# Supplementary material for: Influence of different noninvasive oxygenation support devices on tidal volume
Source: Ann Intensive Care. 2023 Nov 25;13:116. doi: 10.1186/s13613-023-01200-2 (PMC10676331; doi:10.1186/s13613-023-01200-2)
Supplement: Supplementary file 4 — Additional file 4: Table S1. Comparison between the BOUSSIGNAC CPAP and the HELMET CPAP, all mechanics and efforts pooled. Table S2. Active Servo Lung 5000 test lung settings during each experimental setup. Table S3. Characteristics of the Participants (n = 15). [file 13613_2023_1200_MOESM4_ESM.docx]

**Additional file**

**Title:**

**Influence of different noninvasive oxygenation support devices on tidal volume**

**Authors:**

Anne-Fleur HAUDEBOURG*^1,2^ M.D., Tommaso MARAFFI*^1,4^ M.D., Samuel TUFFET^1,2^ M.D., Philippe LE CORVOISIER^5^ M.D., Ph.D., Armand MEKONTSO DESSAP^1,2,3^ M.D., Ph.D., Guillaume CARTEAUX^1,2,3^ M.D., Ph.D.

* AFH and TM contributed equally to the work

**Figure S1: Devices tested.**

All devices were tested on the RespiSim® Manikin connected to an ASL5000 test lung (IngMar Medical, Pittsburg, PA, USA). Care was taken to avoid leaks. A: Non-rebreather oxygen mask (O_2_-mask). B: Boussignac CPAP; C: Helmet CPAP; data from the Boussignac and Helmet CPAP were pooled and analyzed as a whole (CPAP). D: High-flow oxygen through nasal cannula (HFNC). E: NIV using an oro-nasal mask (Mask-NIV); F: NIV using a helmet (Helmet-NIV).

**Figure S2:** **Representation of the experimental setup.**

Each device was tested using the RespiSim® Manikin connected to an ASL5000 test lung (IngMar Medical, Pittsburg, PA, USA). Flow was recorded using a pneumotachograph inserted between the manikin and the test lung. “Mouth pressure was recorded using a differential pressure transducer inserted into the manikin mouth. All signals were recorded using an analog/numeric data-acquisition system (MP150, Biopac systems, Goleta, CA, USA) and stored in a computer for subsequent analysis with AcqKnowledge software (Biopac systems, Goleta, CA, USA).

**Figure S3: Main signals analysis: representative tracings from respiratory cycles during CPAP and Mask-NIV.**

Flow was recorded using a pneumotachograph inserted between the manikin and the test lung. Mouth pressure was recorded using a differential pressure transducer inserted into the manikin mouth. Tidal volume was defined as the area under the positive flow curve (hatched area). The inspiratory time (a) was defined as the time during which the flow was positive. Peak inspiratory flow (b) was the maximum flow recorded during inspiration. Peak mouth pressure (c) was the extreme value (positive or negative) of mouth pressure during inspiratory time. PEEP (d) was measured as the mean pressure recorded during the last 200ms of the expiration at the manikin mouth. The inspiratory mouth pressure-time product (PTPmouth) was the area under the mouth pressure curve from the onset of the simulated inspiratory effort to the end of the inspiratory time (dotted area).

**Table S1: Comparison between the BOUSSIGNAC CPAP and the HELMET CPAP, all mechanics and efforts pooled.**

| **Variable** | **BOUSSIGNAC CPAP** | **Helmet CPAP** | **p** |
| --- | --- | --- | --- |
| Tidal Volume, mL | 258 [153 – 622] | 269 [155 – 596] | 0.820 |
| Peak Inspiratory flow, mL/sec | 430 [262 – 914] | 445 [261 – 881] | 0.570 |
| Positive end-expiratory pressure, cmH_2_O | 9.9 [9.8 – 10.3] | 10.7 [10.3 – 11.0] | 0.129 |
| Mouth Pressure-Time Product, cmH_2_O.sec/cycle | -0.7 [-2.0 - -0.5] | -0.7 [-2.5 - -0.5] | 0.734 |
| Inspiratory mouth pressure swing, cmH_2_O | -1.2 [-2.8 - -0.7] | -1.1 [-3.7 - -0.8)] | 0.496 |

Values are reported as median [25-75 interquartile range]

*CPAP: continuous positive airway pressure.*

**Table S2: Active Servo Lung 5000 test lung settings during each experimental setup.**

| Mechanics and Effort combination | Compliance (mL/cmH_2_O) | Resistances (cmH_2_O/L/sec) | Inspiratory Pmus (cmH_2_O) | Expiratory Pmus (cmH_2_O) | Respiratory rate (breaths per minute) | Inspiratory Rise Time (%)* | Inspiratory Hold (%)* | Inspiratory Release Time (%)* | Expiratory Rise Time (%)* | Expiratory Hold (%)* | Expiratory Rise Time (%)* |
| --- | --- | --- | --- | --- | --- | --- | --- | --- | --- | --- | --- |
| **Normal – Low effort** | 60 | 5 | 5 | 0 | 25 | 30 | 0 | 15 | 0 | 0 | 0 |
| **Normal – Moderate effort** | 60 | 5 | 10 | 0 | 25 | 30 | 0 | 15 | 0 | 0 | 0 |
| **Normal – Distress** | 60 | 5 | 20 | 10 | 25 | 30 | 0 | 5 | 5 | 55 | 5 |
| **Obstructive – Low effort** | 60 | 20 | 5 | 0 | 25 | 30 | 0 | 15 | 0 | 0 | 0 |
| **Obstructive – Moderate effort** | 60 | 20 | 10 | 0 | 25 | 30 | 0 | 15 | 0 | 0 | 0 |
| **Obstructive – Distress** | 60 | 20 | 20 | 10 | 25 | 30 | 0 | 5 | 5 | 55 | 5 |
| **Restrictive – Low effort** | 30 | 5 | 5 | 0 | 25 | 30 | 0 | 15 | 0 | 0 | 0 |
| **Restrictive – Moderate effort** | 30 | 5 | 10 | 0 | 25 | 30 | 0 | 15 | 0 | 0 | 0 |
| **Restrictive - Distress** | 30 | 5 | 20 | 10 | 25 | 30 | 0 | 5 | 5 | 55 | 5 |

*: percentage of the total respiratory cycle time (2.4 seconds in all conditions)

*Pmus: muscle pressure*

**Table S3**: **Characteristics of the Participants (n = 15)**

| Age, year | 31 [29 – 33] |
| --- | --- |
| Male, n (%) | 13 (87) |
| Body mass index, kg/m^2^ | 24 [22 – 24] |
| Baseline characteristics (with oxygen mask) | |
| Tidal volume, mL | 644 [571 – 764] |
| Tidal volume, mL/kg PBW | 8.8 [7.8 – 10.2] |
| Respiratory rate, breaths/min | 11 [9 – 15] |
| Minute ventilation, L/min | 6.6 [5.7 – 10.8] |
| Anterior ventilation, % | 47 [43 – 51] |
| Discomfort scale | 2 [1 – 3] |

*PBW: predicted body weight*.
